# Innate Immunity Evasion by Enteroviruses Linked to Epidemic Hand-Foot-Mouth Disease

**DOI:** 10.3389/fmicb.2018.02422

**Published:** 2018-10-08

**Authors:** Yuefei Jin, Rongguang Zhang, Weidong Wu, Guangcai Duan

**Affiliations:** ^1^Department of Epidemiology, College of Public Health, Zhengzhou University, Zhengzhou, China; ^2^Department of Occupational and Environmental Health, School of Public Health, Xinxiang Medical University, Xinxiang, China

**Keywords:** innate immunity evasion, enteroviruses, coxsackieviruses, hand-foot-mouth disease, type I IFN signaling

## Abstract

Enterovirus (EV) infections are a major threat to global public health, and are responsible for mild respiratory illness, hand, foot, and mouth disease (HFMD), acute hemorrhagic conjunctivitis, aseptic meningitis, myocarditis, severe neonatal sepsis-like disease, and acute flaccid paralysis epidemic. Among them, HFMD is a common pediatric infectious disease caused by EVs of the family *Picornaviridae* including EV-A71, coxsackieviruses (CV)-A2, CV-A6, CV-A10, and CV-A16. Due to lack of vaccines and specific antiviral therapeutics, millions of children still suffer from HFMD. Innate immune system detects foreign invaders by means of a relatively limited number of sensors, such as pattern recognition receptors (PRRs) [e.g., retinoic acid-inducible gene I (RIG-I)-like receptors (RLRs), Toll-like receptors (TLRs), and NOD-like receptors (NLRs)] and even some secreted functional proteins. However, a range of research, highlighted in this review, suggest that EV-associated with HFMD have evolved different strategies to avoid detection by innate immunity *via* different proteases (e.g., 2A, 3C, 2C, and 3D). Ongoing efforts to better understand virus–host interactions that control innate immunity and then distill how that influences HFMD development promises to have real-world significance. In this review, we address this complex topic in nine sections including multiple proteins associated with PRR and type I interferon (IFN) signaling. Recognizing how EVs linked to HFMD evade host innate immune system, we also describe the interactions between them and, finally, suggest future directions to better inform drug development and public health.

## Introduction

Hand, foot, and mouth disease (HFMD) is a common pediatric infectious disease caused by enteroviruses (EVs) of the family *Picornaviridae* including EV-A71, and coxsackieviruses (CV)-A2, CV-A6, CV-A10, and CV-A16 (Ho et al., 1999; Solomon et al., 2010; Centers for Disease Control and Prevention [CDC], 2012) (see **Table 1**). Although usually self-limiting, HFMD can lead to severe complications associated with neural infection or fatal respiratory disease (Chang et al., 1998; Lum et al., 1998). Outbreaks that occurred in Malaysia (1997), Taiwan (1998), Vietnam (2011), and Cambodia (2012) led to 702 child deaths (Ho et al., 1999; Chan et al., 2000; Nguyen et al., 2014; Duong et al., 2016). From 2008 to 2017, accumulated incidence and deaths caused by HFMD in mainland China were approximately 14 million and 3.6 thousand, respectively. Inactivated EV-A71 vaccines in mainland China have been demonstrated to be safe in the target population (infants and young children) and confer a high protective rate against EV-A71 infection-related HFMD (Li et al., 2014). However, to date, millions of children across Asia-Pacific countries still suffer from HFMD every year (Koh et al., 2018).

**Table 1:** Summary of *Enteroviruses* associated with HFMD.

Pathogens	Epidemic country/district	Reference
EV-A71	Japan (1970s); Hong Kong (1985); Australian (1986); Malaysia (1997); Taiwan (1998); Mainland China (2008)	Schmidt et al., 1974; Samuda et al., 1987; Gilbert et al., 1988; Ho et al., 1999; Zhang et al., 2010
CV-A2	Mainland China (since 2008)	Yang et al., 2016
CV-A6	Finland (2008); Singapore (2008); Japan (2011); United States (2011, 2012); Mainland China (2013); France (2010); India (2012)	Blomqvist et al., 2010; Wu et al., 2010; Centers for Disease Control and Prevention [CDC], 2012; Fujimoto et al., 2012; Gopalkrishna et al., 2012; Mirand et al., 2012; Hu et al., 2015
CV-A8	Mainland China (2013); Thailand (2012)	Puenpa et al., 2014; Chen L. et al., 2016
CV-A10	Finland (2008); Mainland China (2008–2012, 2009–2011, 2013–2014, 2015); India (2009–2010)	Blomqvist et al., 2010; Gopalkrishna et al., 2012; Lu Q.B. et al., 2012; He et al., 2013; Chen et al., 2017; Yao et al., 2017
CV-A16	Canada (1957); Australia (1991); England and Wales (1994); Taiwan (2002–2003); Singapore (2002, 2005, and 2007); Vietnam (2005); India (2009); Mainland China (2007, 2009)	Sickles et al., 1955; Robinson et al., 1958; Ferson and Bell, 1991; Bendig and Fleming, 1996; Van Tu et al., 2007; Chang, 2008; Ang et al., 2009; Zou et al., 2012; Kar et al., 2013; Zhu et al., 2013


**FIGURE 1:**
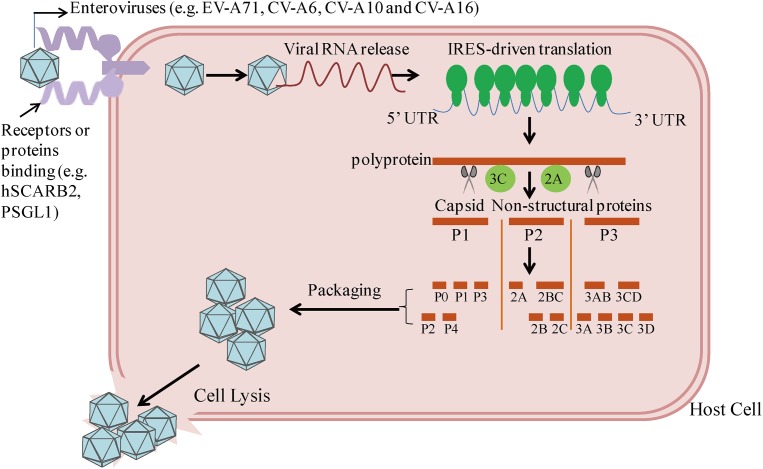
Host cell entry and replication by enteroviruses linked to the HFMD epidemic. By binding with receptors or proteins (e.g., hSCARB2, PSGL-1), enteroviruses enter into host cells, followed by viral RNA release. Cap-independent IRES-driven translation produces a single polyprotein followed by proteolytic cleavage into partially processed products and 11 mature products. Among them, VP1-VP4 leads to the packaging of the capsid.

Enteroviruses are small RNA virus identified by a single-stranded, positive-polarity RNA genome of approximately 7.5 kb in size. The viral capsid consists of 60 identical protomers and each contains four different structural proteins, VP1–VP4. By binding the cell surface receptors human scavenger receptor B2 (hSCARB2) and human P-selectin glycoprotein ligand-1 (PSGL-1), EVs invade host cells and subsequently release viral nucleic acids (Yamayoshi et al., 2009; Solomon et al., 2010; Plevka et al., 2012; Lin et al., 2013). As shown in **Figure 1**, EVs can take advantage of internal ribosome entry site (IRES)-driven translation to subvert host translation machinery (Lee et al., 2017). At different stages, the cleavage of P1–P3 by 2A and 3C proteases results in the synthesis of the capsid proteins VP1–VP4, which subsequently leads to the packaging of the capsid, and seven non-structural proteins: 2A–2C and 3A–3D (Solomon et al., 2010; Plevka et al., 2012). Both sets of these precursors and mature proteins are actively involved with the viral lifecycle, including defense against host innate immunity.

Innate immunity serves as the first line of defense against foreign and dangerous material. Generally, most microbial invaders can be detected and killed within minutes or hours by the body’s defense mechanisms of innate immunity, which do not depend on expansion of antigen-specific lymphocytes but a limited number of secreted proteins and cell-associated receptors (Jensen-Jarolim, 2013). There are at least three broad strategies used by the innate immune system to recognize invading microorganisms (Turvey and Broide, 2010). In the first, innate immunity is equipped with pattern recognition receptors (PRRs) to recognize “microbial non-self” conserved molecular structures [e.g., peptidoglycan, lipopolysaccharide, viral single-stranded RNA (ssRNA) and double-stranded RNA (dsRNA), and viral DNA] termed pathogen-associated molecular patterns (PAMPs). A second fashion equipped by the innate immune system is to monitor dangerous immunologic molecules termed damage-associated molecular patterns (DAMPs) representing common metabolic consequences of infection and inflammation. DAMPs (e.g., unmethylated CpG DNA or pathogen-derived DNA) are upregulated and released during the cell lysis and tissue damage that occurs during infectious and sterile inflammation. For the third strategy, innate immune receptors detect “missing self” molecules expressed by normal healthy cells but not by infected cells or microbes. Recognition of these molecules indicates that “everything is normal,” and an inhibitory signal is followed to suppress activation of the immune response against host tissues.

Enteroviruses have evolved and developed different strategies to evade the innate immune system to facilitate replication inside the host cell. There are abundant studies concerning host innate immunity evasion by EVs. In the present review, we mainly focus on the innate immune evasion by EVs associated with HFMD epidemic.

## Blockade of PRR Signaling Cascades and Type I IFN Signaling

Pattern recognition receptors residing in membranes and the cytosol of innate immune cells such as macrophages, plasmacytoid dendritic cells (pDCs), and dendritic cells (DCs) have evolved to recognize foreign components essential to microbial pathogenicity (Akira et al., 2006). Several classes of PRRs, including retinoic acid-inducible gene I (RIG-I)-like receptors (RLRs), Toll-like receptors (TLRs), and NOD-like receptors (NLRs), are responsible for inducing the production of type I IFNs that are important innate immune regulators during viral infections (Akira et al., 2006). Thus far, three members of RLRs have been identified: RIG-I, MDA5, and Laboratory of Genetics and Physiology 2 (LGP2) which mainly recognize viral dsRNA (Yoneyama et al., 2004). So far, 13 mammalian TLRs have been described, expressed either on the cell surface or in the endosomal compartments. Among them, cell surface TLRs (e.g., TLR1, TLR2, TLR4, TLR5, TLR6, and TLR11) sense lipids, lipoproteins, or peptidoglycans from bacteria, fungi, or protozoa. Endosomal TLR3, TLR7/8, and TLR9 detect viral dsRNA, ssRNA, and endogenous DNA, respectively (Baccala et al., 2007). Recognition of bacterial proteins and viral RNA in the cytoplasm by some NLRs leads to the assembly of inflammasomes followed by the activation of inflammatory caspases. The best known inflammasome-forming NLRs are NLR family pyrin domain-containing protein-1 (NLRP1), NLRP3, and NLR family caspase recruitment domain-containing protein-4 (NLRC4) (Broz and Dixit, 2016). Activation of the above immune receptors controls production of type I interferon (IFN), IFN-α/β, that are secreted by many cell types following a viral infection and can cause neighboring cells to express genes with potential antiviral effects (Muller et al., 1994). Pretreatment with type I IFN can suppress EV-A71 and CV-A16 infection *in vivo* (Yang et al., 2015; Sun et al., 2016), which can be explained by effective strategies employed by EVs to avoid and/or attenuate production of IFN-α/β and thus their effects on immune responses. Taken together, blockade of signaling by PRR provides a key strategy for evasion of innate immunity employed by EV-A71 and CV-A16.

### Inhibition of RIG-I Activation

**FIGURE 2:**
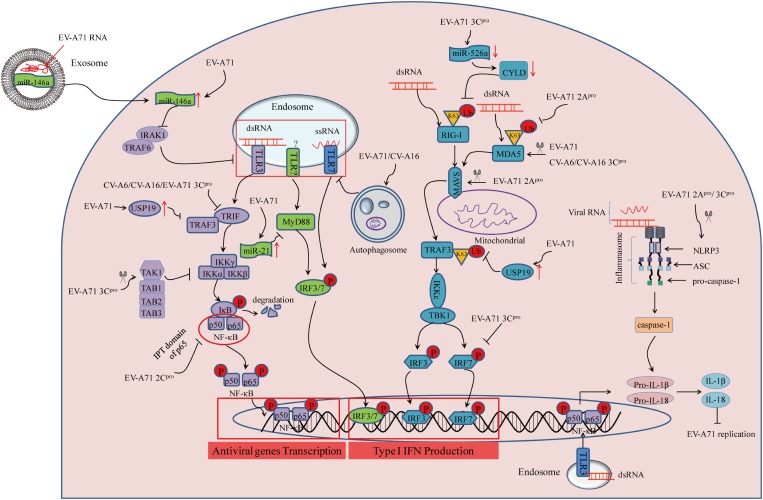
Summary of PRR-mediated innate immunity during enterovirus infection linked to the HFMD epidemic Three cytosolic signaling pathways inhibited by viral infection are represented, as follows: (1) TLR activation leads to signaling through TRIF, TRAF3, and IKKα/β to turn on NF-κB-p50/p65 nuclear transport, or MyD88-mediated IRF3/7 activation; (2) RIG-I and MDA5 activation requires binding to dsRNA and subsequent K63-linked ubiquitination. This signals through mitochondrial-bound MAVS, leading to TBK1/IKKε activation to initiate activity of the transcription factors IRF3/7; (3) assembly of NLRP3 inflammasome requires the sensor NLRP3, the adaptor protein ASC, and pro-caspase-1. Recognition of viral RNA leads to NLRP3 activation, which subsequently results in inflammatory caspase-1activation and IL-1β/IL-18 secretion.

Retinoic acid-inducible gene I-I is an intracellular dsRNA sensor. After recognizing viral dsRNA, it undergoes conformational alterations and post-translational modification including K63-linked ubiquitination on lysine residues of the CARD and C-terminal domains, and further regulates type I IFN-mediated host antiviral innate immunity (Maelfait and Beyaert, 2012). EV-A71 infection inhibits type I IFN signaling by downregulating RIG-I ubiquitination in human rhabdomyosarcoma (RD) cells. However, upregulation of RIG-I ubiquitination by transfection with HA-Ub vector increases expression of IFN-β and IFN-stimulated genes (ISGs) after EV71 infection (Chen N. et al., 2016). In another study, it was found that EV-A71 3C protein (3C^pro^) can inhibit IFN-β expression by targeting the adaptor RIG-I in 293T cells transfected with vectors that can increase amounts of 3C^pro^ and RIG-I (Lei et al., 2010). MicroRNAs (miRNAs) have critical roles in regulating virus-host interactions (Cui et al., 2010). Previous studies suggested that the ubiquitination status of RIG-I is regulated by CYLD, a tumor suppressor originally identified as a genetic defect in familial cylindromatosis (Bignell et al., 2000). Downregulation of miR-526a by EV-A71 3C^pro^ impairs RIG-I-mediated type I IFN production through IRF7 cleavage, and downregulation of CYLD in THP-1 cells, while ectopic miR-526a expression inhibits the EV71 replication (Xu et al., 2014). At the same time, pretreatment with *all-trans* retinoic acid (ATRA) provides antiviral effects through enhancing RIG-I signaling pathway in human monocytic cell line U937 (Chen et al., 2014). However, the mechanism by which ATRA affects RIG-I signaling remains unclear. Together, the lines of evidence described above suggest that inhibition of RIG-I activation by 3C^pro^ provides a strategy of innate immune evasion employed by EV-A71 (see **Figure 2**).

### Inhibition of MDA5 Activation

MDA5, a member of the RLRs family, senses intracellular dsRNA, has a similar structure as RIG-I (Yoneyama et al., 2004). EV-A71 infection enhances MDA5 degradation, and over-expression of MDA5 can reverse the suppression of type I IFN transcription (Kuo et al., 2013). For further study, Feng et al. (2014) suggested that EV-A71-derived 2A protein (2A^pro^) counteracts the antiviral type I IFN response by cleaving MDA5 in infected cells. Similar to RIG-I, enhancing K63-linked ubiquitination by ARRDC4 results in activation of the downstream innate signaling pathway of MDA5 during EV-A71 infection (Meng et al., 2017). ARRDC4 functions as an adapter, recruiting ubiquitin-protein ligases to their specific substrates. But unlike EV-A71, 3C^pro^ of CV-A16, and CV-A6 is responsible for the disruption of MDA5 in 293T cells transfected with relative vectors that can increase amounts of 3C^pro^ (Rui et al., 2017). Therefore, inhibition of MDA5 activation provides a novel strategy equipped by EV-A71, CV-A16, and CV-A6 to escape host antiviral innate immunity (see **Figure 2**).

### Inhibition of Mitochondrial-Associated Signaling Molecules

Mitochondrial antiviral-signaling adaptor protein (MAVS) serves as a key adaptor in cellular antiviral innate immunity, residing on the outer membranes of the mitochondria and peroxisomes. MAVS is best known to initiate TANK-binding kinase 1 (TBK1)-dependent and nuclear factor-kappa B (NF-κB)-dependent antiviral gene transcription (Seth et al., 2005). EV-A71 2A^pro^ was confirmed to suppress interferon regulatory factor (IRF)3 signaling through the cleavage of MAVS, resulting in IFN-α/β reduction in HeLa cells (Feng et al., 2014). Meanwhile, another study revealed that EV-A71 infection caused morphologic and functional changes of the mitochondria, whereby *in vitro* cleavage assay indicated that EV-A71 approached MAVS and led to MAVS cleavage by 2A^pro^ (Wang et al., 2013). Combining the results from the above two publications provides a good explanation for the immune evasion mechanisms employed by EV-A71. For studies of CV-A16 and CV-A6, evidence suggested that the association of adaptor MAVS and MDA5 was disrupted by 3C^pro^ in a dose dependent manner in 293T cells transfected with plasmids encoding MDA5-N-Myc, MAVS-Flag, and HA-3C (Rui et al., 2017). In considering the above results, it is believed that EV-A71, CV-A16, and CV-A6 share a common immune evasion mechanism by inhibiting MAVS activation (see **Figure 2**). However, it remains to be definitively shown whether there is direct or indirect interaction between MAVS and these viruses.

### Inhibition of TLR-Dependent Antiviral Signaling

TLR signaling is essential to induce type I IFN production during EV-A71 infection (Wang et al., 2016). To escape host innate immunity, EV-A71 3C^pro^ has been found to block IFN-β production through the adapter protein TIR-domain-containing adapter-inducing interferon-β (TRIF) in response to endosomal TLR3 activation (Lei et al., 2011). Similar to TLR3, TLR7 also resides on endosomes to sense foreign viral ssRNA. Increased EV-A71 and CV-A16 replication induced by autophagy leads to the degradation of endosomes, which further suppresses TLR7-mediated type I IFN responses in human bronchial epithelial (16HBE) cells by using autophage inhibitor (3-MA) and laser confocal (Song et al., 2018). Likewise, upregulation of miR-21 upon EV-A71 infection can suppress myeloid differentiation factor 88 (MyD88) downstream of TLRs, which subsequently blocks type I IFN-mediated antiviral responses *in vitro* with miR-21 inhibitor and transfection of miRNA and siRNA (Feng et al., 2017). In addition, CV-A16 and CV-A6 3C^pro^ are proposed to subvert host innate immune responses by suppressing TLR-mediated NF-κB signaling in 293T cells transfected with NF-κB-luc promoter reporter and Flag-TLR3 expression vector alone or with CV-A16 3C^pro^ or CV-A6 3C^pro^ or their protease mutation H40D (Rui et al., 2017). Therefore, direct or indirect inhibition of TLR-dependent antiviral signaling are effective mechanisms of immune evasion employed by EV-A71, CV-A16, and CV-A6 (see **Figure 2**).

### Inhibition of NLRP3 Inflammasome Activation

The NLRP3 inflammasome complex consists of pro-caspase-1 (casp1), ASC, and NLRP3. Activation of the NLRP3 inflammasome leads to the secretion of interleukin (IL)-1β and IL-18 to provide host protective antiviral effects (Broz and Dixit, 2016). *In vivo* experiments suggest that pretreatment with recombinant IL-18 can reverse EV-A71 infection-induced pathogenesis (Li et al., 2017). EV-A71 interferes with inflammasome assembly through the cleavage of NLRP3 by 2A^pro^ and 3C^pro^ in 293T cells transfected with wild-type NLRP3 or NLRP3 mutants. Meanwhile, 3C^pro^ of EV-A71 interacts with NLRP3 and suppresses IL-1β secretion (Wang H. et al., 2015). By contrast, EV-A71-derived 3D protein (3D^pro^) binds with NLRP3 to facilitate the assembly of inflammasome complexes, which results in the secretion of IL-1β in 293T cells transfected with plasmids encoding pro-IL-1β, Flag-pro-caspsase-1, Flag-NLRP3, Flag-ASC, and EV-A71 3D^pro^ (Wang W. et al., 2017). Together, EV-A71 is able to suppress NLRP3 inflammasome activation as a mode of immune evasion (see **Figure 2**). Nonetheless, additional studies are warranted to elucidate inconsistent results regarding IL-1β production and determine the mechanism of IL-18 secretion during EV-A71 infection.

### Inhibition of IRF Activation

Interferon regulatory factor-mediated expression of type I IFN and IFN-inducible genes plays a central role in responses to viral infection (Janeway and Medzhitov, 2002). EV-A71 can suppress ISG expression by blocking IRF3 activation in HeLa cells (Lei et al., 2011). Another study revealed that EV-A71 3C^pro^ was responsible for the blockade of IRF3 activation and IFN-α/β production in 293T cells transfected with GFP-IRF3 alone or along with Flag-3C^pro^ (Lei et al., 2010). However, Lei et al. (2013) found that EV-A71 3C^pro^ was required to cleave IRF7 rather than IRF3 to delay the type I IFN response *in vitro* with transfection of GFP-3C^pro^ alone, or IRF7 and GFP-3C^pro^ along with IFN-β-Luc. Regardless, these lines of evidence suggest that inhibition of IRF activation is an effective mechanism of immune evasion by EV-A71 (see **Figure 2**).

### Antagonizing IFN Signaling and Jak/STAT Signaling

**FIGURE 3:**
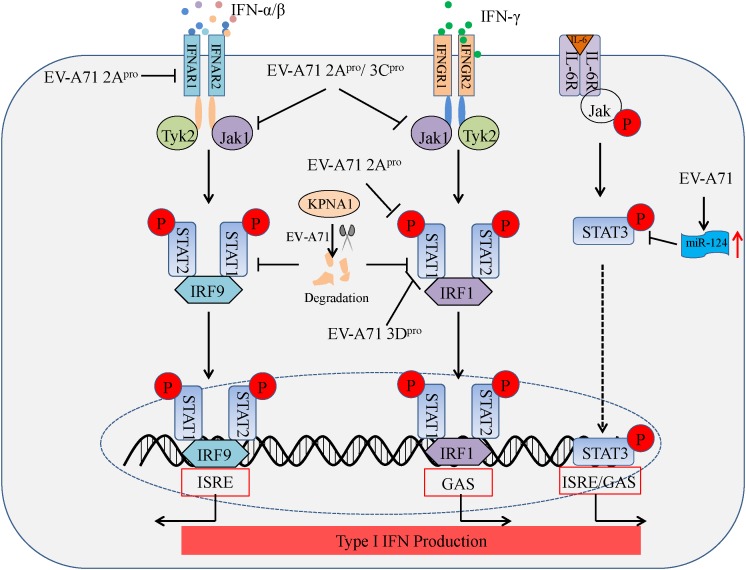
Summary of IFN signaling and Jak/STAT signaling suppressed by enteroviruses linked to the HFMD epidemic. Binding of IFNAR by type I IFN (IFN-α/β) triggers downstream kinases, Jak1 and Tyk2. Jak1 and Tyk2 phosphorylate STAT1 and STAT2, which leads to the formation of the STAT1–IRF9–STAT2 complex. This complex translocates to the nucleus and initiates transcription of IFN-stimulated response elements (ISREs) in specific genes. Likewise, binding of IFNGR by type II IFN (IFN-γ) triggers downstream kinases, Jak1 and Tyk2. Jak1 and Tyk2 phosphorylate STAT1 and STAT2, which leads to the formation of the STAT1-IRF1-STAT2 complex. This complex translocates to the nucleus and initiates transcription of IFN-γ-activated sequences (GAS) in additional genes. Nuclear accumulation of phosphorylated STAT3 (p-STAT3) depends on the binding of IL-6R and IL-6, whereby p-STAT3 also initiates transcription *via* ISRE/GAS elements.

Type I IFNs (IFN-α/β) and type II IFN (IFN-γ) form the first line of defense against viral infection, and also play a critical role in immunosurveillance for malignant cells. The binding of IFN-α/β and IFN-γ to their specific receptors, type I IFN receptors (IFNAR1 and IFNAR2) and type II IFN receptors (IFNGR1 and IFNGR2), leads to the rapid autophosphorylation and activation of Janus-activated kinase (Jak)/signal transducer and activator of transcription (STAT) pathways which in turn regulates IFN-α/β transcription (Darnell et al., 1994). EV-A71 2A^pro^ blocks STAT1, STAT2, Jak1, and Tyk2 phosphorylation by reducing IFNAR1 expression in 293T cells transfected with 2A^pro^ (Lu J. et al., 2012). In another study, EV-A71 2A^pro^ attenuated IFN-γ-induced serine phosphorylation of STAT1 by blocking ERK signaling in mouse embryonic fibroblasts (MEFs) transfected with 2A^pro^ along with IFN-γ treatment, while EV-A71 3D^pro^ attenuation of IFN-γ signaling was accompanied by a STAT1 decrease in MEFs transfected with 3D^pro^ (Wang C. et al., 2015). Furthermore, EV-A71-induced miR-124 can antagonize the antiviral activity of STAT3 (Chang Z. et al., 2017). Based on the above findings, nearly all studies to date provide consistent evidence that EV-A71 2A^pro^ and 3C^pro^ act as antagonists of cellular type I IFN signaling. In addition, Liu et al. (2014) found that EV-A71 can also inhibit the type I antiviral pathway by downregulating Jak1 expression *in vitro*, while IFNAR1 expression does not significantly change in infected cells. Likewise, EV-A71 suppresses IFN-β production by blocking Jak/STAT signaling through degradation of karyopherin-α1 (KPNA1), a nuclear localization signal receptor for p-STAT1 *in vitro*, although this appears to occur independently of EV-A71 2A^pro^ and 3C^pro^ (Wang C. et al., 2015). These reports suggest EV-A71-mediated inhibition of IFN signaling and Jak/STAT activation suppresses type I IFN production (see **Figure 3**). However, controversies remain, and more studies will be needed to reveal the precise mechanisms of IFN signaling and Jak/STAT signaling inhibition during EV-A71 infection.

### Inhibition of TRAF3 Activation

Tumor necrosis factor receptor-associated factor (TRAF) 3 is a crucial adaptor molecule for TLR3- and RLR-mediated type I IFN signaling (Kawai and Akira, 2006; Loo and Gale, 2011). It has been suggested that EV-A71 infection induces ubiquitin-specific protease 19 (USP19) gene expression, which negatively regulates type I IFN signaling by suppressing TRAF3 ubiquitination of K63-linkage in 293T cells transfected with Flag-TRAF3, HA-USP19, and HA-Ub (Gu et al., 2017). This evidence indicates that EV-A71 can escape host innate immunity by suppressing TRAF3 ubiquitination *via* USP19 induction (see **Figure 2**). In future studies, elucidating the upstream molecular regulators of TRAF3 will be critical to gain a more complete understanding of the mechanisms of EV-A71 infection-induced innate immune evasion. In addition, targeted inhibition of USP19 may be useful for the treatment of EV-A71 infection-associated HFMD.

### Inhibition of NF-κB Activation

The NF-κB p65/p50 heterodimer is the most abundant signaling complex of the NF-κB family, and plays a key role in host defense against viral infection (Rahman and McFadden, 2011). Nuclear transport of p65/p50 heterodimers promotes the secretion of cytokines and chemokines, which are significant for host defense against viral infection through innate immunity (Fagerlund et al., 2005). As reported (Du et al., 2015), by interacting with the IPT domain of p65, EV-A71 2C^pro^ suppresses the formation of p65/p50 heterodimers in 293T cells transfected with 2C^pro^ and truncation constructs of p65, which provides another novel strategy employed by EV-A71 to escape innate immunity (see **Figure 2**). In addition, NF-κB activation can also be inhibited by EV-A71 3C^pro^ through cleavage of the transforming growth factor-l-activated kinase 1 (TAK1) complex, a TAK1/TAK1-binding protein 1 (TAB1)/TAB2/TAB3 complex, in 293 cells transfected with relative plasmids (Lei et al., 2014). Whether EV-A71 infection affects degradation of NF-κB inhibitor-α (IκBα) as a mechanism to inhibit the NF-κB pathway is essential to be identified in the future.

## Other Signaling Pathways

ZAP, a mammalian host restriction factor, has been demonstrated to suppress RNA virus replication (Zhang et al., 2007). EV-A71 3C^pro^ was found to induce ZAP cleavage, while over-expression of ZAP can inhibit EV-A71 replication in 293T cells with ZAP transfection (Xie et al., 2018). EV-A71 can control innate immunity by regulating miRNA functions (Ho et al., 2014; Fu et al., 2017). For example, EV-A71 infection upregulates miR-146 expression, which further suppresses TLR signaling and type I IFN production by targeting IL-1 receptor-associated kinase 1 (IRAK1) and TRAF6 in RD cells transfected with the miR-146a over-expressing vector (Ho et al., 2014). The high level miR-146 can be from exosomes secreted by EV-A71-infected cells, which is packaged with exosomal viral RNA, and that in turn facilitates EV-A71 replication in the recipient cells by suppressing type I IFN response (see **Figure 2**; Fu et al., 2017). IFN-induced dsRNA-activated protein kinase R (PKR), an IFG, acts as a PRR recognizing dsRNA. A short N-terminal PKR fragment originates from PKR cleavage mediated by EV-A71 3C^pro^ can enhance EV-A71 replication in RD cells transfected with the vector, increasing concentrations of plasmids encoding the PKR (1–188) or PKR (1–188) K64E fragment. Therefore, inhibition of ZAP, miR-146a induction, or PKR cleavage may represent another mechanism equipped by EV-A71 to escape host antiviral responses. These studies provide clues for the design of therapeutic strategies against EV-A71 infection by targeting ZAP, miR-146, and PKR.

## Conclusion and Perspectives

Enterovirus infections continue to pose a significant public health threat worldwide, and are associated with the epidemics of mild respiratory illness, HFMD, acute hemorrhagic conjunctivitis, aseptic meningitis, myocarditis, severe neonatal sepsis-like disease, and acute flaccid paralysis. Among these afflictions, HFMD epidemics are serious public health issues for children from Asia-Pacific countries. However, few vaccines and specific antiviral therapeutics are applied for disease control and clinical practice, because of the unique viral structure and mechanisms of innate immunity evasion. The body of evidence presented above suggests that EVs linked to the HFMD epidemic are equipped with various unique strategies to evade multiple arms of the innate immune response by suppressing intracellular antiviral type I IFN signaling, regulating miRNAs, or by modulating functional protein expression (see **Table 2**). In particular, shielding PRRs recognition becomes a main fashion of innate immunity evasion by 2A, 3C, and 3D proteases derived from EV-A71, CV-A6, or CV-A16. Another mechanism is to block critical intracellular molecules such as NF-κB p65/p50 and ZAP to interfere with antiviral effects. Additionally, more and more studies suggest that miRNAs such as miR-146a, miR-124, miR-21, and miR-526a have a crucial role in modulating innate immune response through PRR signaling and type I IFN signaling. In summary, EVs linked to the HFMD epidemic are relatively efficient at modulating innate immunity, and this property allows these viruses to successfully establish infection in humans.

**Table 2:** Summary of viral proteins and their targets of host proteins or pathways.

Viral proteins	Host proteins/pathways targeted	Reference
EV-A71 3C^pro^	(a) RIG-I/IFN-β (b) miR-526a/K63-linked ubiquitination/RIG-I-mediated type I IFN production (c) TLR3/TRIF/IFN-β (d) NLRP3/IL-1β (e) IRF3/type I IFN response (f) IRF7/type I IFN response (g) TAK1 complex cleavage (h) ZAP cleavage (i) PKR cleavage	Lei et al., 2010, 2011, 2013; Xu et al., 2014; Wang H. et al., 2015; Xie et al., 2018; Wang C. et al., 2015 Lei et al., 2014; Chang Y.H. et al., 2017
EV-A71 2A^pro^	(a) MDA5 cleavage (b) MAVS/IRF3 signaling (c) IFNAR1/Jak/STAT (d) IFN-γ signaling/ the serine phosphorylation of STAT1 (e) NLRP3 cleavage	Lu J. et al., 2012; Wang et al., 2013; Wang L.C. et al., 2015; Wang L.C. et al., 2015; Feng et al., 2014
EV-A71 2C^pro^	p65/p50 heterodimers	Du et al., 2015
EV-A71 3D^pro^	(a) NLRP3/ IL-1β (b) IFN-γ signaling/STAT1	Wang H. et al., 2015; Wang W. et al., 2017
CV-A16 and CV-A6 3C^pro^	(a) MDA5/MAVS (b) MDA5/type I IFN response (c) TLR3/NF-κB	Rui et al., 2017


Recently, many drugs have been developed and showed protective antiviral effects in cellular and mouse models (Hung et al., 2011; Zhang et al., 2013; Lin et al., 2016). However, most of them still remain unknown for clinical practice. Both *in vitro* and *in vivo* studies have indicated that type I IFN administration can suppress EV-A71 replication and increase survival rate (Liu et al., 2005; Yi et al., 2011). A multicenter, randomized, double-blind clinical trial suggested that IFN-α2b spray could rapidly relieved symptoms of HFMD (Lin et al., 2016). Collectively, these layers of evidence indicate that type I IFN should be candidate drug for clinical treatment in the near future. As mentioned earlier, ATRA and ARRDC4 provide antiviral effects through enhancing RIG-I signaling and MDA5 signaling during EV-A71 infection *in vitro* and *in vivo* (Chen et al., 2014; Meng et al., 2017). Due to lack of clinical experiment, we cannot say rashly that ATRA and ARRDC4 are useful for the real-world clinical scenarios, but this evidence justifiably matter most to clinicians. Based on above evidence, the earlier event of type I IFN reduction is because of the interactions of viral proteases with PRR signaling or functional proteins. Synthesis of 2A, 3C, and 3D inhibitors should be useful for HFMD treatment. An *in vitro* study found that combination of 3C inhibitors and IFN-α have synergistic effects on EV-A71 replication (Hung et al., 2011). Another study suggested that siRNAs targeting the 2A region of the EV-A71 genome exerted antiviral effects *in vitro* (Liu et al., 2016). However, the other viral proteases still can reduce type I IFN production. Therefore, understanding the structural roles of 2A, 3C, and 3D proteases will provide crucial information for the design of a broad spectrum inhibitor. Regulation of miRNAs expression is additional developing therapeutic strategies for HFMD. Synthesis of miR-146a, miR-21, and miR-124 inhibitors and miR-526a intervention will be beneficial for HFMD treatment in the future.

Although previous studies have made significant progress in obtaining new knowledge of the interplay between EVs linked to the HFMD epidemic and innate immunity, several important questions remain to be clarified in the future studies. First, due to minimal evidence suggesting that ssRNA or dsRNA are ligands for PRR stimulation during EVs infection, it is important to identify viral and/or host factors that contribute to the PRR recognition. Second, does mitochondrial damage play any role in RLR-mediated signaling? MAVS residing on outer membrane of mitochondria is downstream of RLR signaling, and a previous study has demonstrated that EV-A71 infection can cause mitochondrial damage (Dang et al., 2017). Therefore, the inhibition of RIG-I, MDA5, and MAVS may be responsible for the disruption of the outer mitochondrial membrane. Third, the mechanism of EV-A71 regulation of NLRP3 assembly is inconsistent to date, and how EV-A71 affects NLRP3 activation and IL-1β secretion should be confirmed in the future. Additionally, the precise targets of EV-A71 in regulating IRF and IFN signaling remain to be fully revealed. Last but not the least, diagnostic meaning of miRNAs during HFMD development should be considered in the future study. As HFMD continues to cause threat to children’s health, identifying the viral factors that antagonize innate immunity will be helpful to develop future therapeutics to restrict the burden of HFMD.

## Author Contributions

YJ collected the references and wrote this manuscript. RZ, WW, and GD critically reviewed and revised the article. All authors contributed to article’s edits and approved the final manuscript.

## Conflict of Interest Statement

The authors declare that the research was conducted in the absence of any commercial or financial relationships that could be construed as a potential conflict of interest.
